# Disease Incidence and Results of Extremity Lesion Treatment: Mersey Region Soft Tissue Sarcomas (1975–1985)

**DOI:** 10.1080/13577149878046

**Published:** 1998-06

**Authors:** Michael J. Jane, Peter J. Hughes

**Affiliations:** Royal Liverpool University Hospital Prescot St Liverpool L7 8XP UK

## Abstract

*Purpose.* The incidence and treatment results of extremity soft tissue sarcoma (STS) in the Mersey Region, in the absence of a Multi-Disciplinary Unit, for the period 1975–1985, have been analysed.

*Subjects and methods.* Data from cases presenting with STS within the Mersey region, from 1 January 1975 until 31 December 1985, were reviewed. Only patients with sarcoma of head and neck, thoracic wall, abdominal wall, retroperitoneum, limb girdle or extremity were included. Extremity lesions were staged according to the MTS system. Pathological data also were assigned a grade according to tumour differentiation, mitosis count and tumour necrosis. Data from patients with a minimum follow-up of 5 years were collated, and patterns of treatment failure were investigated. Finally, time to first occurrence was analysed.

*Results and Discussion.* The incidence of STS in this study was identical to that reported by the US Department of Health in 1976. Five year survival rate for Stage I tumours was only 51.7% which compares very unfavourably with contemporary series from Multi-Disciplinary Units. Five year survival rate following wide local excision ± adjuvant therapy is 52.4%, while that following amputation ± adjuvant therapy is 45.5%. While not attaining the results reported by other centres, limb-sparing surgery does not appear to appreciably prejudice long-term survival.

*Conclusions.* STS are rare in the UK, leading to poor classification and suboptimal treatment of lesions. It is important to establish multidisciplinary teams of surgeons, radiologists, radiotherapists and oncologists to plan and organise multimodality therapy for STS.


*Sarcoma* (1998) **2**, 89–96

ORIGINAL ARTICLE

### Disease incidence and results of extremity lesion treatment: Mersey Region soft tissue sarcomas (1975–1985)

MICHAEL J. JANE & PETER J. HUGHES

*Royal Liverpool University Hospital, Liverpool, UK*

**Abstract**

*Purpose.* The incidence and treatment results of extremity soft tissue sarcoma (STS) in the Mersey Region, in the absence of a Multi-Disciplinary Unit, for the period 1975–1985, have been analysed.

*Subjects and methods.* Data from cases presenting with STS within the Mersey region, from 1 January 1975 until 31 December 1985, were reviewed. Only patients with sarcoma of head and neck, thoracic wall, abdominal wall, retroperitoneum, limb girdle or extremity were included. Extremity lesions were staged according to the MTS system. Pathological data also were assigned a grade according to tumour differentiation, mitosis count and tumour necrosis. Data from patients with a minimum follow-up of 5 years were collated, and patterns of treatment failure were investigated. Finally, time to first occurrence was analysed.

*Results and Discussion.* The incidence of STS in this study was identical to that reported by the US Department of Health in 1976. Five year survival rate for Stage I tumours was only 51.7% which compares very unfavourably with contemporary series from Multi-Disciplinary Units. Five year survival rate following wide local excision ± adjuvant therapy is 52.4%, while that following amputation ± adjuvant therapy is 45.5%. While not attaining the results reported by other centres, limb-sparing surgery does not appear to appreciably prejudice long-term survival.

*Conclusions.* STS are rare in the UK, leading to poor classification and suboptimal treatment of lesions. It is important to establish multidisciplinary teams of surgeons, radiologists, radiotherapists and oncologists to plan and organise multimodality therapy for STS.

### Introduction

Soft Tissue Sarcomas (STS) are rare tumours, accounting for less than 1% of malignant neoplasms.[1] Despite their rarity, recent advances in limb-sparing techniques and the use of adjuvant radiotherapy and chemotherapy[2] in multidisciplinary units[3,4] has seen an improvement in their management. In the Mersey Region between 1975 and 1985 no such specialist unit existed. In this analysis, the incidence and treatment results of extremity soft tissue sarcomas occurring in Mersey between 1975 and 1985 were investigated.

### Subjects and methods

The Mersey Region Cancer Registry database was utilised for this study. The case records of patients presenting with soft tissue sarcomas within the Mersey Region were reviewed from 1 January 1975 to 31 December 1985, inclusive. For eligibility, patients required a histologically proven diagnosis of sarcoma arising from the head and neck, thoracic wall, abdominal wall, retroperitoneum, limb girdle or extremity. Visceral sarcomas and metastatic sarcomas of unknown primary were excluded. The eligible patient population was then examined to derive age/sex distribution and age-specific annual incidence. Patients with limb girdle and extremity STS were further examined to calculate their age/sex distribution data and the relative incidence of histological types of STS.

Extremity lesions were staged according to the MTS staging system, 1980.[5] The pathological reports were reviewed and assigned a grade (high or low) according to the work of Trojani *et al.*[6] This method uses tumour differentiation, mitosis count and tumour necrosis to provide an estimation of grade. The surgical technique used and results of investigation were correlated to allow estimation of surgical stage. Patients with a minimum follow-up of 5 years were identified and methods of primary management were collated. The 5-year survival rates were analysed by primary treatment employed,

Correspondence to: M. J. Jane, Royal Liverpool University Hospital, Prescot St, Liverpool L7 8XP, UK. Tel. + 44 151 7064118; Fax: + 44 151 7065839.

1357-714X/98/020089-08 ©1998 Carfax Publishing Ltd by commonest histological type and by disease stage (MTS Surgical Staging System, 1980). Patterns of treatment failure were then investigated. Finally, time to first recurrence was analysed.

### Results

*Incidence of STS (Mersey 1975–1985)*

The above criteria were satisfied by 544 patients; 309 males and 235 females, giving a male to female ratio for the study of 1.32:1. The mean age of the 309 males was 53.9 years (range 1 month to 97 years). The mean age of the 253 females was 55.5 years (range 9 days to 88 years). The age/sex distribution of STS in the Mersey Region (1975–85) is shown in Fig. 1. The number of new cases of STS diagnosed annually is shown in Fig. 2. The mean number of new cases occurring annually was 49.5 (range 37–66) for all sites. Age-specific annual incidence for STS is shown in Fig. 3. The peak incidence of STS for both sexes occurs in the over 85 years age group, in which STS are twice as common in males as in females. It should be noted that, for both sexes, STS become progressively more common after 45 years of age.

The sites of occurrence of STS for the 544 patients in the study are shown in Table 1. A group of 274 patients with limb girdle and extremity STS were identified. The mean number of new STS occurring annually in the lower and upper limbs was 18.2 (range 12–34) and 6.7 (range 3–13), respectively. The mean number of extremity lesions occurring annually was thus 24.9 (range 18–41). Further age/sex distribution data for extremity lesions were calculated (Table 2) giving a male to female ratio of 1.3:1 and a lower limb to upper limb ratio of 2.7:1.

The relative incidence of histological types of STS of the extremities is presented in Table 3.

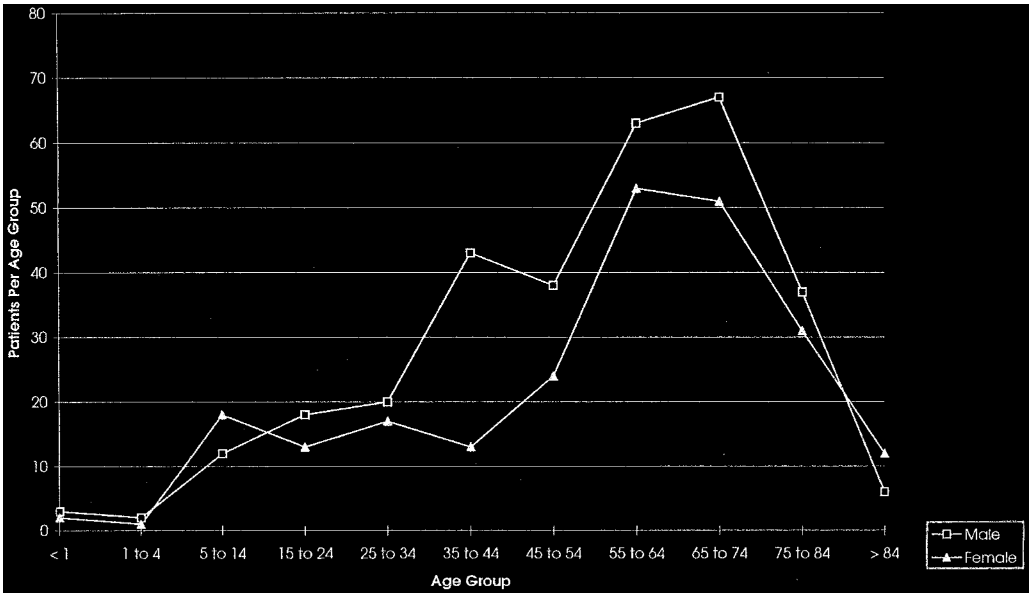

**Fig. 1.** *Age and sex distribution of soft tissue sarcomas (Mersey Region 1975–1985).*

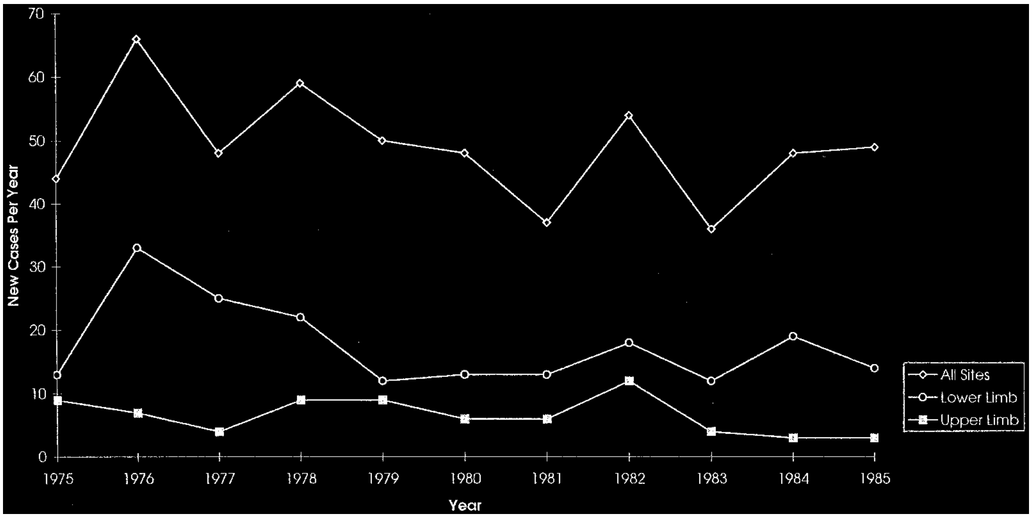

**Fig. 2.** *Incidence of soft tissue sarcomas (Mersey Region 1975–1985).*

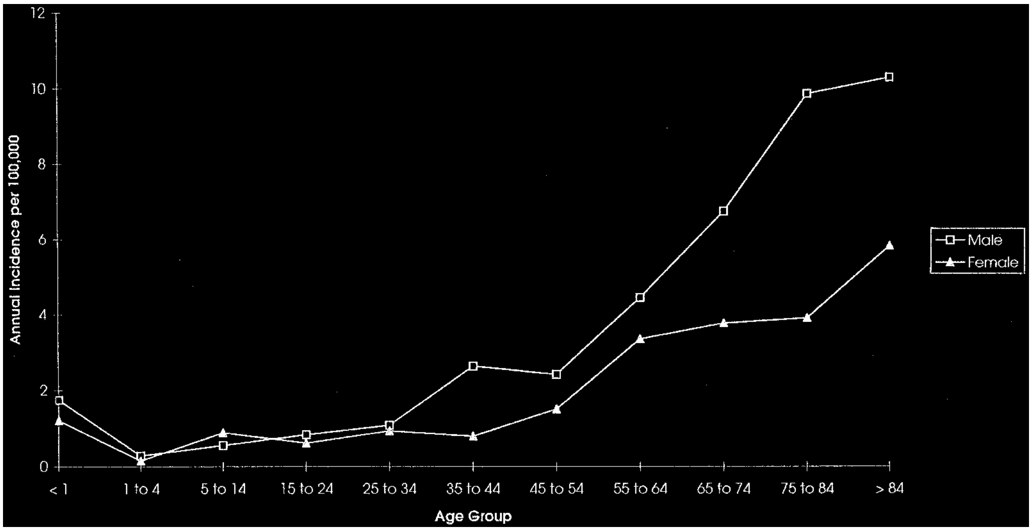

**Fig. 3.** *Age-specific annual incidence of soft tissue sarcomas (Mersey Region 1975–1985).*

**Table 1.** *Sites of occurrence of STS in the Mersey Region (1975–1985)*

| Head and neck (%) | Trunk (%) | Retroperitoneum (%) | Upper extremity*(%) | Lower extremity**(%) |
|---|---|---|---|---|
| 46 (8.5) | 145 (26.7) | 79 (14.5) | 74 (13.6) | 200 (36.8) |

*Shoulder/arm, 44 (8.1); forearm/wrist, 23(4.2); hand, 7 (1.2).
**Gluteal/thigh, 121 (22.2); knee/leg, 56 (10.3); ankle/foot, 23 (4.2).

**Table 2.** *Age/sex distribution—of extremity STS, Mersey Region (1975–1985)*

| | No. of patients | Mean age in years (range) |
|---|---|---|
| Male | | |
| Upper limb | 40 | 50.1 (8–76) |
| Lower limb | 115 | 53.0 (9–92) |
| Female | | |
| Upper limb | 34 | 60.8 (8–97) |
| Lower limb | 85 | 57.2 (8–89) |

**Table 3.** *Relative incidence of histological types of extremity STS*

| | Lower (%) | Upper (%) |
|---|---|---|
| (1) Fibrosarcoma | 56 (20.4) | 22 (8.0) |
| (2) Unclassified | 35 (12.8) | 16 (5.8) |
| (3) Malignant fibrous histiocytoma | 12 (4.4) | 5 (1.8) |
| (4) Liposarcomas (unclassified) | 11 (4.0) | 5 (1.8) |
| (5) Synovial sarcomas | 8 (2.9) | 6 (2.2) |
| (6) Rhabdomyosarcoma | 12 (4.4) | 1 (0.4) |
| (7) Spindle cell sarcoma | 19 (3.6) | 2 (0.7) |
| (8) Myxoid liposarcoma | 11 (4.0) | 0 (0.00) |
| (10) Giant cell sarcoma | 6 (2.2) | 2 (0.7) |
| Others | 29 (10.5) | 14 (5.2) |

### *Results of Extremity Soft Tissue Sarcomas Treatment*

The 274 extremity lesions were staged according to the MTS Staging System, 1980[5] (Table 4). The methods of primary management were collated (Table 5). Nine patients were not treated due to patient refusal, disease being unresectable or patient being deemed medically unfit for treatment. Sixty-eight (24.8%) patients underwent excisional biopsy ± adjuvant therapy. No incisional biopsies were performed and, for the purpose of this study, wide excision was defined as tumour removal with a surrounding cuff of normal tissue greater than 2 cm in all directions. It was not possible to define accu-

**Table 4**. *Surgical stage of 274 extremity lesions*

| Stage | Number | Percentage of total |
|---|---|---|
| IA | 42 | 15.3 |
| IB | 60 | 21.9 |
| IIA | 52 | 19.0 |
| IIB | 92 | 33.6 |
| IIIA | 11 | 4.0 |
| IIIB | 17 | 6.2 |

rately oncological surgical margins (intralesional, marginal, wide or radical) from examination of the surgical records and pathological reports.

Radiotherapy, when utilised, was given post-operatively after 2–3 weeks delay to allow wound healing. Typically, radiotherapy was administered by megavoltage technique using parallel opposed fields, vital structures being protected by wedging the X-ray field. Palliative radiotherapy in one or two fractions was used in some patients, but most patients received their therapeutic external beam irradiation to the primary site, drain tracks and lymph nodes; 35–65 Gy over 3–6 weeks being the usual regime.

Chemotherapy was used as part of the primary management in certain situations, which included patients presenting with disseminated disease and patients with advanced primaries not amenable to surgical treatment and who did not respond to radiotherapy. A variety of chemotherapeutic regimes

**Table 5**. *Influence of primary treatment method on survival in 233 patients with extremity STS followed up for greater than 5 years (Mersey 1975–1985)*

| Treatment | Number Treated | Number surviving 5 years | % |
|---|---|---|---|
| No treatment (unfit or refusing) | 9 | 0 | 0 |
| Excision biopsy | 19 | 2 | 10.5 |
| Excision biopsy + radiotherapy | 20 | 5 | 25.0 |
| Excision biopsy + chemotherapy | 9 | 0 | 0 |
| Excision biopsy + radiotherapy + chemotherapy | 11 | 3 | 27.3 |
| Wide local excision | 98 | 56 | 57.1 |
| Wide local excision + radiotherapy | 35 | 17 | 48.6 |
| Wide local excision + chemotherapy | 3 | 1 | 33.3 |
| Wide local excision + radiotherapy + chemotherapy | 7 | 1 | 14.3 |
| Amputation | 18 | 9 | 50.0 |
| Amputation + radiotherapy | 1 | 0 | 0 |
| Amputation + chemotherapy | 2 | 0 | 0 |
| Amputation + radiotherapy + chemotherapy | 1 | 1 | 100.0 |

**Table 6**. *Five-year survival rates of the 10 most common histological types of STS (201 patients)*

| | No. of cases | No. surviving 5 years | % |
|---|---|---|---|
| Fibrosarcoma | 68 | 28 | 41.2 |
| Unclassified | 51 | 9 | 17.6 |
| Malignant fibrous histiocytoma | 12 | 11 | 91.7 |
| Liposarcomas (unclassified) | 14 | 6 | 42.9 |
| Synovial cell sarcomas | 13 | 6 | 46.2 |
| Rhabdomyosarcoma | 10 | 3 | 30 |
| Spindle cell sarcoma | 10 | 3 | 30 |
| Leiomyosarcoma | 8 | 3 | 37.5 |
| Myxoid liposarcoma | 8 | 5 | 62.7 |
| Giant cell sarcoma | 7 | 3 | 42.9 |

**Table 7**. *Treatment results of 233 cases of extremity STS classified according to stage of disease*

| Stage | Number of cases | Number surviving 5 years | % |
|---|---|---|---|
| Stage I (IA + IB) | 84 | 48 | 57.1 |
| Stage II (IIA + IIB) | 128 | 47 | 36.7 |
| Stage III (IIIA + IIIB) | 21 | 0 | 0 |

**Table 8**. *Patterns of failure by histological type for 233 patients with extremity STS*

| | No. | Local failure alone | Distant metastasis alone | Local failure and metastasis | Lymph nodes | Local Recurrence (%) |
|---|---|---|---|---|---|---|
| Fibrosarcoma | 68 | 15 | 9 | 19 | 6 | 50.0 |
| Unclassified | 51 | 11 | 6 | 11 | 3 | 43.1 |
| Malignant fibrous histiocytoma | 12 | 1 | 3 | 3 | – | 33.3 |
| Liposarcomas (unclassified) | 14 | 2 | 3 | 3 | – | 35.7 |
| Synovial cell sarcomas | 13 | 4 | 2 | 5 | 1 | 69.2 |
| Rhabdomyosarcoma | 10 | 4 | – | 4 | 1 | 80.0 |
| Spindle cell sarcoma | 10 | 3 | – | 6 | 2 | 90.0 |
| Leiomyosarcoma | 8 | 2 | 3 | 3 | 2 | 62.5 |
| Myxoid liposarcoma | 8 | 3 | 1 | 1 | – | 50.0 |
| Giant cell sarcoma | 7 | 3 | 1 | 1 | – | 57.1 |
| Overall for all 233 STS | | 55 (23.6%) | 31 (13.3%) | 65 (27.9%) | 18 (7.7%) | |

were used during the study period including adriamycin plus DTIC and methotrexate, vincristine, adriamycin and cyclophosphamide, and high-dose methotrexate alone and with vincristine. These were all administered i.v., no use was made of hyperthermic isolation perfusion. No patients received adjuvant chemotherapy.

The 5-year survival rates for 233 patients with a minimum follow up of 5 years were analysed by method of treatment employed (Table 5). The overall 5-year survival rate for the series was 40.8%. The amputation rate was 9.4%. The 5-year survival rate of patients treated by wide local excision ± adjuvant was 52.4%. This compares with the 5-year survival rate of 45.5% for those patients treated by amputation ± adjuvant therapy. Limb-sparing surgery did not appear to prejudice long-term survival. Excision biopsy alone is to be condemned as a method of treatment as this is associated with only 10.5% 5-year survival.

The 5-year survival rates occurring in the 10 most common histological types were calculated (Table 6). The results of treatment by disease stage (MTS Surgical Staging System, 1980) were also investigated (Table 7).

The patterns of failure of 233 patients were then studied. Lymph node involvement occurred in 18 (6.6%) patients. Local failure occurred in 120 (43.8%). Distant metastases without local recurrence occurred in 31 (11.3%). Treatment failure by histological type is shown (Table 8).

Finally, in this study, time to first local recurrence in patients was analysed. Seventy percent of tumours that recurred locally did so by the first year, 95% recurred by 3 years, and only two first local recurrences occurred more than 5 years from primary surgery.

### Discussion

In this study the incidence of STS in Mersey Region was investigated. At 2.0 per 100 000 (2.36/100 000 for males and 1.68 per 100 000 for females) this rate is identical to the rate reported by the US Department of Health in 1976.[1] The incidence of STS increases steadily with age after 34 years, and is higher in males, such that in the greater than 85-year age group STS are twice as common amongst men. This increase with age may be due to environmental exposure to carcinogens, cumulative degenerative genetic damage and immunosuppression of old age. Other studies have shown an increased occurrence of those tumours amongst men.[7] In this study males out-numbered females by 1.32 to 1. The reason for this is unknown but may be due to occupational exposure to carcinogens.

When compared with previous studies,[8–11] the relative incidence of histological types of STS show some marked disparities. Unclassified tumours are far more common in this series (19.7% of cases compared with 10.5%, a mean of four series). This may be a reflection of the lack of expertise in pathology, as appropriate biopsy technique and histological experience can enable more tumours to be categorised. Synovial sarcomas and rhabdomyosarcomas occurred with a reduced frequency when compared to other studies. Fibrosarcomas comprised the largest group of tumours, being 23.3% of the total. Malignant fibrous histiocytoma was diagnosed rather infrequently (6.6%). This may again be related to pathological inexperience, as many tumours formally regarded as fibrosarcomas are now classified as malignant fibrous histiocytomas. Electron microscopy and the use of special stains have enabled greater categorisation of STS. This is clearly a reason for the establishment of STS Pathology Units.

The distribution of STS was largely as reported in previous studies.[8–11] Retroperitoneal tumours were more common than expected (14.5% of cases compared with a mean of 11.6% in four other studies), and lower limb tumours were less common than expected (36.8% compared with a mean of 41.8%).

Thus, data from the Mersey study appears consistent with previous investigations, and the few results appearing outside the expected range appear to be due to inexperience in histological diagnosis.

### *Extremity STS*

In this study, lower limb lesions outnumbered upper limb lesions by 2.7 to 1. This probably reflects the greater soft tissue bulk of the lower limb. Females developing tumours were older than their male counterparts; females with upper limb tumours were, on average, 4.2 years older than their male patients. Anatomically, STS were distributed such that 44.2% of extremity lesions occurred in the buttock/thigh region, knee and lower leg tumours comprised 20.4% of lesions, and shoulder and arm tumours comprised 16% of all lesions. Soft tissue sarcomas at other more distal sites were relatively rare. This overall distribution compares well with previous studies.

Examination of histological types in extremity lesions confirmed the preponderance of fibrosarcomas and unclassified STS together with the diminished incidence of malignant fibrous histiocytomas. It was noted that synovial sarcomas are increased in incidence (2.8% for all sites, 5.1% for extremity lesions) and leiomyosarcomas are less common in the extremities (7.2% of all sites; 4.0% for extremity lesions).

The 274 extremity lesions were staged according to the Enniking MTS surgical staging system, 1980. This form of retrospective cross-correlating between surgical technique, pathological examination, preoperative examination and investigation is well known to be inaccurate. Better surgical techniques along oncogenic lines will allow better future evaluation of data; however, within these constraints the distribution of extremity lesions was as follows: stage Ia 15.3%, Ib 21.9%, IIa 19.0%, IIb 33.6%, IIIa 4.0% and IIIb 6.2%. This compares well to the work of Simon and Enniking who examined a large series of extremity STS.[12]

There are several interesting observations to be made upon the management of extremity STS in the Mersey Region. Firstly, the amputation rate at 9.9% is low and compares favourably with contemporary series.[3,13] Excision biopsy alone was performed in 22 cases, 23 patients received excision biopsy and radiotherapy. There is abundant evidence to show that excision biopsy alone is an inadequate form of treatment. There appears to be no logical explanation why so many patients treated by excision biopsy were not subsequently given radiotherapy. Surgeons may not have referred these patients or, conversely, radiotherapists may not have considered these tumours radio-responsive. Again, in the case of wide local excision, 41 patients had supplementary radiotherapy whilst 115 patients had surgery alone. If the results are examined the 5-year survival rate for wide local excision alone was 57.1% compared with 48.6% for wide local excision plus radiotherapy. This may imply that radiotherapy was reserved for patients with larger tumours resulting in poorer prognosis. The 5-year survival rate for Mersey extremity STS (40.8%) appears low when contemporary series of patients treated by limb-sparing surgery are attaining 61% (range, 47.6–67.0%) 5-year survival rates.[14–16] Solutions to explain this poor outcome cannot be derived from these data due to the lack of comparable groupings.

The present study reveals that 5-year survival rate following wide local excision ± adjuvant therapy is 52.4%, and that following amputation ± adjuvant therapy it is 45.5%. The present study therefore reveals that limb-sparing surgery, as performed in the Mersey Region, whilst not attaining the results of other centres,[17] does not appear to compromise long-term survival when compared to amputation.

Five-year survival by histopathological type and stage of disease at presentation was also reviewed. Better differentiated tumours were noted to have better 5-year survival rates. The unclassified group, as would be expected, had the poorest prognosis, and the 5-year survival rate was only 17.6%. Five-year survival by disease stage was as follows: stage I 51.7%, stage II 36.7%, stage III 0%. Contemporary studies show 90–95% 5-year survival rates for stage I lesions, 45% survival for stage II lesions and 5% survival for stage III lesions.[14,15] Most of these contemporary series have patients with similar stage disease but differ in that aggressive radiotherapy was used either pre-or post-operatively.

The patterns of failure of patients treated for STS reveal some interesting features. Patients developing distant metastases alone amounted to 13.3%, but most published series show isolated distant metastasis is the commonest mode of treatment failure. The present study reveals local failure ± distant metastasis occurred in 51.9% of patients. Contemporary series showed this mode of failure occurs in only 16.0%. The relationship between local control and subsequent distant metastasis and tumour-related mortality is exceedingly complex.[18] The marked rate of local recurrence in this series cannot be linked causally to distant metastasis. It does, however, suggest inadequate treatment in the management of local disease.

Lymph node metastasis occurred in 18 patients (7.6%) treated during the study period; this agrees well with the 5.8% lymph node involvement rate reported by Ariel.[19] Metastasis to lymph nodes occurred with increasing frequency in patients with leiomyosarcomas, synovial cell sarcomas and fibrosarcomas, in comparison with other series.

The local recurrence rate for excision biopsy alone was found to be 81.8%; this compares to a local recurrence rate of 47.8% when local excision is supplemented with radiotherapy. The addition of radiotherapy to wide local excision decreases the local recurrence rate from 35.7 to 29.3%. Local recurrence rate for patients treated by amputationwas 18.5%; this compares favourably with reported local failure rates of 15.8% for patients treated by amputation.[12,20,21] Unfortunately there was no way of categorising amputees as being marginal, wide or radical. Definite conclusions about what constitutes adequate local treatment cannot be drawn from this work due to the likely heterogeneous nature of both the patient groups and treatment protocols. Patients who develop local recurrence are clearly at increased risk of developing distant metastasis and tumour-related mortality. There was under-use of radiotherapy in the Merseyside Region during the study period, this cannot explain the poor survival rates reported here, but may provide an explanation for the very high and undesirable rates of local recurrence. This strengthens the argument for a combined modality treatment approach to the management of STS.

Histological type also appears to influence local recurrence. Spindle cell sarcomas, rhabdomyosarcomas and synovial cell sarcomas had high local recurrence rates at 90.0, 80.0 and 69.2%, respectively. Wide variation in these rates have previously been noted.[22] Local recurrence rates for fibrosarcomas, liposarcomas and leiomyosarcomas were 50.0, 35.7 and 62.5%, respectively. It is well known that certain histological types show a predeliction to local recurrence. However all these rates are high when compared with other studies.[12,23] Clearly the local control was inadequate with regard to all histological types. The time to local recurrence was in accord with the work of Cantin *et al.*[24] and revealed that of these tumours destined to recur 95% had done so by 5 years.

In summary, this investigation has shown the Mersey Region population is at average risk of developing STS. The rarity of these lesions mean that in the UK, a given surgeon with an average sized practice will only see an STS every 1–2 years. This inexperience has lead to suboptimal treatment of extremity lesions in terms of both local recurrence and 5-year survival. This inexperience is also manifest in pathological assessment, as there were clear weaknesses in classification of the lesions during the study period. This work has further highlighted the importance of establishing multidisciplinary teams involving surgeons, radiologists, radiotherapists and oncologists to plan and organise multimodality therapy for STS.

### References

1 Cancer Patient Survival. Report No. 5 US Department of Health, Education and Welfare Publication No. (NIH) 77–992, 1976.
2 Svoboda VH, Krawczyk J. Management of soft tissue sarcomas in Portsmouth 1965–1985. *Clin Oncol (R Coll Radiol)* 1991; 3:162–7. May.
3 Watson DI, Coventry BJ, Langlois SL, *et al.* Soft Tissue Sarcoma of the extremity. Experience with limb sparing surgery. *Med J Australia* 1994; 160:412–6.
4 Wilkund T, Huuhtanen R, Blomqvist C, *et al.* The importance of a multidisciplinary group in the treatment of Soft Tissue Sarcomas. *Eur J. Cancer* 1996; 32:269–73.
5 Enniking WF, Spaner SS, Godman MA. A system for the surgical staging of musculoskeletal sarcoma. *Clin Orthop Rel Res* 1980; 153:106–20.
6 Trojani M, Contesso G, Coindre JM. Soft tissue sarcomas of adults; Study of pathological prognostic variables and definition of a histopathological grading system. *Int J Cancer* 1984; 33:37–42.
7 Storm HH. Cancers of the soft tissues. *Cancer Surveys* 1994; 19–20:197–217.
8 Potter DA, Glenn J, Kinsella T, *et al.* Patterns of recurrence in patients with high grade Soft Tissue Sarcoma. *J Clin Oncol* 1985; 3:353–366.
9 Suit HD. Patterns of failure after treatment of sarcoma of soft tissue by radical surgery or by conservative surgery and radiation. *Cancer Treat Symp* 1983; 2:241–6.
10 Romsdahl MM, Lindberg RD, Martin RG. Surgery and post operative radiotherapy in the treatment of Soft Tissue Sarcoma in adults. *Am J Roentgenol Radiat Ther Nucl Med* 1975; 123:123–9.
11 Russel WO, Cohen J, Enzinger FM. A clinical and pathological staging system for soft tissue sarcomas. *Cancer* 1977; 40: 1562–70.
12 Simon MA, Enneking WF. The Management of soft tissue sarcomas of the extremities. *J Bone Joint Surg* 1976; 58:317–23.
13 Berlin O, Stener B, Angervall L, *et al.* A multivariate analysis of the 6 to 26 year prognosis in 137 patients. *Acta Orthop Scand* 1990; 61:475–86.
14 Karakousis CP, Emrich LJ, Rao U. Selective combination of modalities in soft tissue sarcomas: Limb salvage and survival. *Semin Surg Oncol* 1988; 4:78–81.
15 Guo-Hui LI, Jin-Qing LI, Yong-Hui CAI. Surgical management of soft tissue sarcomas, with an analysis of 313 cases. *Semin Surg Oncol* 1988; 4:82–5.
16 Suit HD, Mankin HJ, Wood WC. Treatment of patients with stage Mo soft tissue sarcomas. *J Clin Oncol* 1988; 6:854–62.
17 National Institutes of Health Consensus Development Panel on Limb sparing treatment of adult Soft Tissue Sarcomas and Osteosarcomas. Introduction and conclusions. *Cancer Treat Symp* 1985; 3:71–81.
18 Gustafson P, Rooser B, Rydholm A. Is there no influence of local control on the rate of metastases in high grade soft tissue sarcoma? *Cancer* 1990; 65:1727–9.
19 Ariel IM. Incidence of metastases to lymph nodes from soft tissue sarcomas. *Semin Surg Oncol* 1988; 4:27–9.
20 Shui MH, Castro EB, Hajdu SI. Surgical treatment of 297 soft tissue sarcomas of the lower extremity. *Ann Surg* 1975; 182: 597–602.
21 Markhede G, Angervall L, Stener B. A multivariate analysis of prognosis after surgical treatment of malignant soft tissue tumours. *Cancer* 1982; 49:1721–33.
22 Rosenberg SA, Suit HD, Baker LH. Sarcomas of soft tissues. In: DeVita VT (ed) *Cancer—Principles and Practice of Oncology, 2nd ed.* Philadelphia, PA: JB Lippincott Co.
23 Gustafson P. Soft tissue sarcoma. Epidemiology and progress in 508 patients. *Acta Orthop Scand Suppl* 1994; 259:1–31.
24 Catin J, McNeer GP, Chu FC. The problem of local recurrence after treatment of soft tissue sarcoma. *Ann Surg* 1968; 168:47–53.
